# Molecular Detection and Genotyping of *Toxoplasma gondii* in Chicken, Beef, and Lamb Meat Consumed in Northwestern Iran

**Published:** 2017

**Authors:** Mahmoud MAHAMI-OSKOUEI, Mohammad MORADI, Esmaeil FALLAH, Faezeh HAMIDI, Najibeh ASL RAHNAMAYE AKBARI

**Affiliations:** 1. Immunology Research Center, Tabriz University of Medical Sciences, Tabriz, Iran; 2. Dept. of Parasitology and Mycology, Faculty of Medicine, Tabriz University of Medical Sciences, Tabriz, Iran

**Keywords:** *Toxoplasma gondii*, Surface antigen P22, Meat, PCR, Iran

## Abstract

**Background:**

*Toxoplasma gondii*, the obligate, intracellular parasite afflicts human in diverse ways such as ingestion of tissue cysts in undercooked meat. The aim of this study was to assess the prevalence and genotyping of *Toxoplasma* isolated from meat samples being consumed in East Azerbaijan, Northwest of Iran.

**Methods:**

Overall, 150 samples including chicken, beef and lamb meat were collected from retailers in different regions of Tabriz, northwestern Iran during April to September 2014. Molecular detection was done by amplifying B1 gene and *T. gondii* surface antigen 2 (SAG2) loci. For genotyping of *T. gondii,* restriction fragment length polymorphism (RFLP) was performed using *Sau*3AI and *Hha*I restriction enzymes. Finally, three positive isolate from each host was sequenced to evaluate and phylogenetic analysis.

**Results:**

Overall, 26 (17.33%) samples were positive for *T. gondii* including 4 (8%) isolate from chicken, 8 (16%) isolates from cattle and 14 (28%) isolates from sheep. According to the RFLP patterns, sequencing and phylogenetic results, all of the samples were identified as genotype I.

**Conclusion:**

The results indicated a potential risk of transmission of the disease through the consumption of infected meat. This is particularly important especially for pregnant women and immuno-compromised patients and this suggests that the training on the prevention of infection is necessary.

## Introduction

*T. gondii* an important etiologic agent of toxoplasmosis and foodborne diseases is now a global concern (1). A human can normally infect via two major ways i.e. exposure to contaminated soil by oocyst, or ingestion of uncooked or under-cooked meat containing tissue cyst (2, 3). Toxoplasmosis has variable manifestations in the host ranging from asymptomatic to serious symptoms (4). Pathogenesis of the disease in human depends on host factors (immunity, genetic, nutrition) and parasite factors (inoculums size, parasite life-cycle stage, strain) (5–7). The most severe clinical outcomes are observed in immunocompromised patients such as human immunodeficiency virus (HIV) and children born with congenital toxoplasmosis (8, 9).

Different genes loci and markers have been evaluated and utilized for identification and classification of *T. gondii*, however, SAG2 gene locus by restriction fragment length polymorphism (RFLP) technique have been widely used for this purpose (10). *T. gondii* has been analyzed into three genotypes (I, II, III) based on RFLP (11). Molecular characterization and genotyping of *T. gondii* in different populations and regions may not only help to study prevalence and identification of dominant *T. gondii* genotypes, determine the most common source and way of transmission, potential drugs towards it and production of vaccine, as well as control of the disease (11, 12).

In consideration to the prevalence in Iran, 4.7% meat has been the reported as the possible source (13). Genotyping of 13 positive soil samples in Iran showed that 69% were type III, 3 isolates depicted type I and III (23%), while 8% were type I (14). The genotyping of toxoplasmosis only on meat products and blood samples of animal were focused (15–17) and the dominant genetic type in positive meat products has been found as type I (18–20).

Given the importance of toxoplasmosis especially in seronegative pregnant women and immunocompromised patients as well as the relatively high consumption of meat in diets and the risk of transmitting the infection, this study was designed to investigation the prevalence and genotyping of *Toxoplasma* isolated from meat samples in East Azerbaijan, Northwest of Iran.

## Materials and Methods

### Sample collection

A total of 150 samples, included chicken, beef and lamb meat (50 samples of each) were collected from retailers randomly selected in different regions: North, South, East, West and Center of Tabriz, East Azerbaijan Province during April to September 2014. Of 50 g of each sample was separated and stored at −20 °C until testing.

### Sample preparation and DNA extraction

About 20 g of each meat sample mixed with 15 ml of saline containing 0.05% pepsin, and then well homogenized, was incubated at 37 °C for 4 h and centrifuged at 6000 rpm for 5 min. The supernatant was discarded and DNA was extracted from the pellet by using a commercial kit (Genet Bio Global Gene Network, South Korea) following the manufacturer’s instructions. The extracts were stored at −20 °C for further use.

### Molecular detection by B1 gene

Polymerase chain reaction by amplifying B1 gene was used for detection of *Toxoplasma* in all samples. The specific primers were used describing in previous studies (14, 21): forward (5′-GGAACTGCATCCGTTCATGAG-3′) and reverse (5′-TCTTTAAAGCGTTCGTGGTC-3′). PCR volume was adjusted to 20 μl including: Tris-HCL (pH 9.0)10 mM, each dNTP 250 μM, KCL (30mM), MgCl_2_ (1.5mM), Taq DNA polymerase (1U), 10 pmol each primer and 2μl (∼25ng) of template DNA. PCR was set up at 95 °C for 10 min as initial denaturation followed by 40 cycles of denaturation 94 °C (30 s), annealing 55 °C (1 min), extension 72 °C (1 min) and elongation step at 72 °C (10 min) as final extension. Finally, 5μl of amplified PCR products were electrophoresed on 2% agarose gel and visualized under UV after staining with safe stain dye.

### SAG2 locus polymerase chain reaction

SAG2 locus of *Toxoplasma* was selected and nested-PCR method was used for amplifying this gene. Primers were used for 5′-SAG2 primary PCR (forward: 5′-GCTACCTCGAACAGGAACAC-3′, reverse: 5′-GCATCAACAGTCTTCGTTGC-3′), 5′-SAG2 secondary amplification (forward: 5′-GAAATGTTTCAGGTTGCTGC-3′, reverse: 5′-GCAAGAGCGAACTTGAACAC-3′) and also 3′-SAG2 primary PCR (forward: 5′-TCTGTTCTCCGAAGTGACTCC-3′, reverse: 5′-TCAAAGCGTGCATTATCGC-3′), 3′-SAG2 secondary amplification (forward: 5′-ATTCTCATGCCTCCGCTTC-3′, reverse: 5′-AACGTTTCACGAAGGCACAC-3′) (10). The first round PCR was performed in total volume 20 μl including: Tris-HCL (pH 9.0)10 mM, each dNTP (dATP, dCTP, dGTP, dTTP) 250 μM, KCL (30mM), MgCl_2_ (2mM), Taq DNA polymerase (1U), 20 pmol each primer and 4μl (∼50ng) of template DNA. PCR cycles were set up for 5 min at 94 °C, followed by 41 cycles of 94 °C for 45 sec, 59 °C for 45 sec and 72 °C for 1 min, with a final extension step at 72 °C for 10 min. Second round PCR was done on the amplified PCR product with similar conditions but with annealing temperature set at 54°C and 2 μl of first round product. The amplicons were electrophoresed on 2% agarose gel and visualized under UV. PCR was conducted with positive and negative controls.

### Genotype analysis

Three types (I, II or III) of *T. gondii* were determined by PCR–RFLP of the amplified *SAG2* gene according to previous study (11). For genotyping, the secondary nested-PCR products of 5′-SAG2 and 3′-SAG2 were digested by *Sau*3AI and *Hha*I restriction enzymes respectively (22, 23). To prepare the RFLP reactions, 7μl of PCR product was mixed with 1μl of restriction enzyme and 1μl of enzyme buffer and total volume was adjusted to 15μl by adding distilled water. The preparation was then incubated at 37 °C for 20 min. Restriction fragments were analyzed on after electrophoresis on 3% agarose gel.

### SAG2 sequencing and phylogenetic analysis

In order to evaluate and confirm the results of RFLP, secondary PCR products of nine positive isolates (three of each host) were sequenced by ABIPRISMTM 3130 genetic analyzer automated sequencer. The sequences were compared with GenBank sequences by using Blast (http://blast.ncbi.nlm.nih.gov/Blast.cgi) for sequences similarity and genotype was determined. Multiple sequence alignment was done by (http://multalin.toulouse.inra.fr/multalin). MEGA 5.05 software and maximum likelihood algorithm with kimura 2-parameter model and 1000 bootstrap resampling were used for phylogenetic analysis.

### Statistical analysis

Descriptive statistics were used to describe the data and results were expressed as a percentage.

## Results

**Fig. 1:**
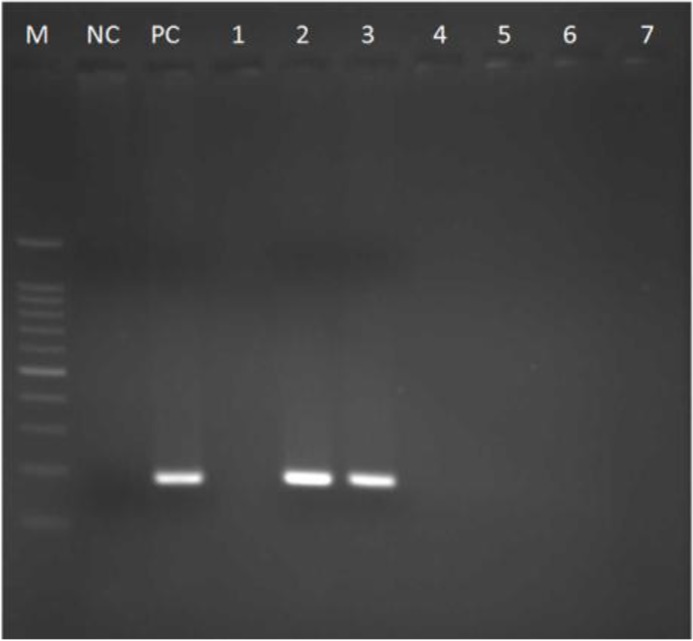
Agarose gel electrophoresis of *T.gondii* B1 gene amplification, M: DNA size marker 100 bp (Fermentas); NC: Negative control; PC: Positive control; Lane 1,4,5–7 negative samples; Lane 2,3 (194 bp), positive meat samples

We obtained 150 samples consisting 50 chicken, 50 beef, and 50 lamb meats. Primarily, *Toxoplasma* in samples was detected by amplifying B1 gene that positive samples showed 194bp band on 2% agarose gel (Fig. 1).

**Fig. 2:**
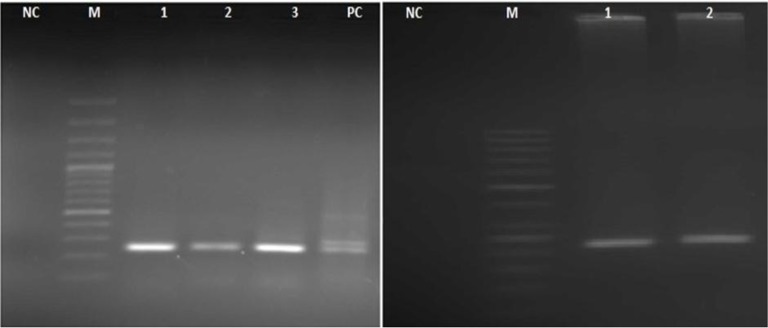
Nested-PCR amplicons of *T.gondii* SAG2 loci. Left (5′-SAG2): NC: Negative control; M: DNA size marker 100 bp; lane 1,2,3 (241 bp): positive meat samples; PC: Positive control. Right (3′-SAG2): NC: Negative control; M: DNA size marker 50 bp; lane 1, 2 (221 bp): positive meat samples

5′ and 3′ flanking of *T. gondii* surface antigen P22 was amplified successfully for all samples by nested-PCR method. After gel electrophoresis of secondary PCR products, we detected 241bp and 221bp fragments for 5′ and 3′ SAG2, respectively (Fig. 2). In total 26 (17.33%) tissue, samples were positive for *T. gondii,* which included 4 (8%) chicken meat, 8 (16%) cattle sample and 14 (28%) sheep tissue.

**Fig. 3:**
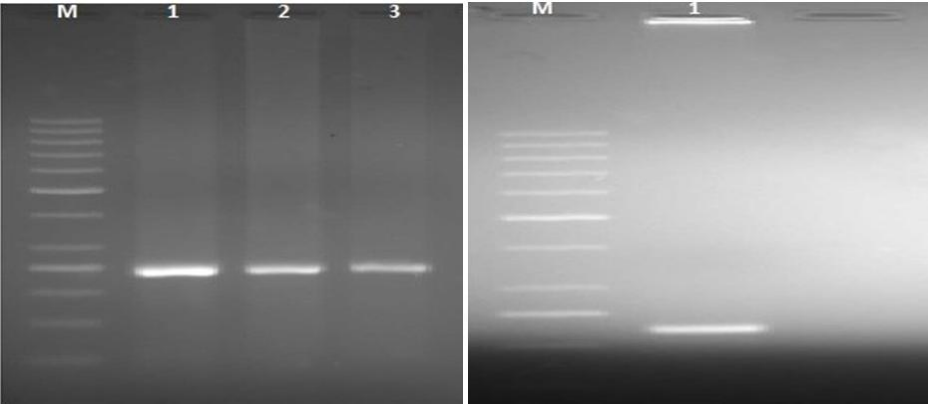
Restriction analysis of 5′-SAG2 locus of *T.gondii* using *Sau*3AI (left): Lane 1, 2, 3 (241 bp): positive meat samples (genotype I) and 3′-SAG2 locus by *Hha*I (right): lane 1 (221 bp): positive meat sample (genotype I); M: DNA size marker 50 bp

All of the 26 positive meat samples were genotyped using 5′ and 3′-SAG2 locus by using RFLP method and two restriction enzymes. These samples showed a 241bp fragment in 5′-SAG2 locus and 221bp fragment in 3′-SAG2, respectively (Fig. 3). According to the RFLP patterns, there was not cutting site on both fragments for these enzymes which indicated all of the samples were of genotype I (100%) based on the previous studies (14, 15) neither genotype II nor III was found in our study.

**Fig. 4:**
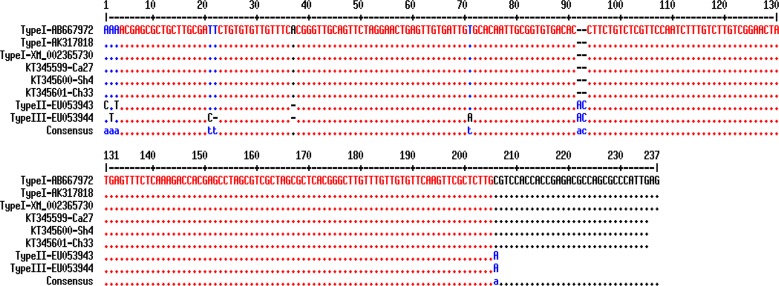
Multiple sequence alignments of *T.gondii* surface antigen 2 gene based on reference sequences. (KT345599, KT345600, KT345601): accession numbers of sequences registered in GenBank from the present study; Ca: Cattle; Sh: Sheep; Ch: Chicken

**Fig. 5:**
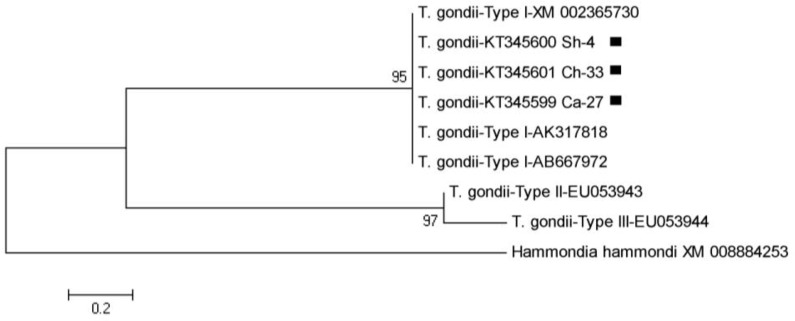
The phylogeny tree of *Toxoplasma gondii* based on SAG2 gene sequences from three different host isolates of Northwest Iran (KT345599-KT345601) and other previous GenBank registered sequences using maximum likelihood algorithm with kimura 2-parameter model and 1000 bootstrap resampling. *Hammondia hammondi* was considered as outgroup branch

The products of the second step of nested-PCR from nine different isolates were selected for sequencing. These nucleotide sequences showed 100% homology in comparison with the other reported sequences in GenBank and after alignment, there was confirmed that *T. gondii* belonged to type I genotype. Three nucleotide sequences (one of each host) from this investigation were submitted and registered in GenBank under following accession numbers KT345599, KT345600, and KT345601. Multiple sequence alignment showed the similarities and differences between these sequences and other GenBank registered sequences (Fig. 4). In phylogenic analysis, *T. gondii* isolates of this study relied on a branch which represents type I and similar to sequences that submitted from other countries. For drawing the phylogeny tree, *Hammondia Hammond* (XM_008884253) was considered as an outgroup (Fig. 5). R (Transition/Transversion bias) was estimated to 0.55 using kimura 2-parameter model in MEGA 5.05.

## Discussion

Toxoplasmosis is one of the major causes of life-threatening infection especially in immuno-compromised patients such as HIV positive and patients undergoing anti-cancer chemotherapy or others who received immunosuppressive drugs (8, 9, 24). From the epidemiological point of view, meat is important source for transmission of this parasite (25–27). The high prevalence of toxoplasmosis among domestic animals such as cattle, sheep, and chicken (28, 29) and raw or undercooked meat can be an important factor in the transmission of the infection to humans (2, 3).

Utilized surface antigens of *T. gondii* was considered as appropriate antigens for detection and genotyping (10), we also used SAG2 loci and nested-PCR method for this purpose in the present investigation. Detection of *T. gondii* in tachyzoite or bradyzoite form is not very difficult because the parasite gets destroyed in a short time (25) but oocyst form, which has a resistant wall consisting of several layers and is difficult for DNA extraction (8, 10, 30). Identification and genotyping of *T. gondii* directly from tissue have some advantage since preventing artificial prevalence of strains (18, 31–32).

Present investigation was conducted to detect and genotyping of *T. gondii* in chicken, beef and lamb meat samples from East Azerbaijan, Northwest Iran. The main objective of this study was to identify *T. gondii* in meat consumption. In our study, 17.33% of all samples were positive by using B1 gene and SAG2 loci. The highest and lowest rate of infection was furnished in sheep and chicken tissues, respectively. However, RFLP pattern, sequencing and phylogenetic results showed that type I was predominant genotype of *T. gondii.*

In the previous study, which performed for genotyping of *T. gondii* in meat products in the northwest of Iran, parasite contamination found in 16.6, 19.1, 15 and 56.6% (overall 24.3%) in salami, sausages, hamburger and kebab samples, respectively and genotype I was reported as the most prevalent genotype based on SAG2 and RFLP method (15). Our results were incompatible with this study.

In another study from southwest of Iran, performed by amplification of GRA6 using nested-PCR method, found high contamination infection rate of *T. gondii* in sheep (38%), nevertheless almost dissimilar rate in cattle samples (8.57%). However, they did not genotype the parasite (33). The high level of contamination in sheep seems to be related to sheep rearing conditions in the region. Investigation from Ahvaz west of Iran used B1 gene to determine the prevalence of *T. gondii* in meat and meat products and showed 14% and 4% positivity in lamb and beef samples, respectively (13). Similar study conducted in UK, depicted 38% of meat samples (including beef, lamb, and pork) *T. gondii* contamination.

After SAG2 analysis, all samples were of type I along with simultaneous presence of SAG2 types (I, II) (5). Although the lack of other genotypes in this study is consistent with the previous studies (34, 35), some studies have also found type II or III as the main genotypes (17, 29, 36). These controversial results are probably due to various genotypes of *T. gondii* in different geographic regions. *Toxoplasma* strains in animals and human have not the same distribution in a same geographical region.

## Conclusion

A potential risk of transmission of the disease indicated through the consumption of infected meat. This is particularly important especially for pregnant women and immuno-compromised patients and this proposes that control and skillful training are required for the prevention of infection. Given the importance of this parasite in terms of public health, further studies should be designed to understand the actual rate and kind of genotypes of *T. gondii* in other hosts and sources.
